# Preparation and Properties of *Camellia oleifera* Fruit Shell Cellulose Nanoparticle Material for Selective Enrichment of Histidine Proteins From Foods

**DOI:** 10.1155/ijfo/6353950

**Published:** 2026-05-22

**Authors:** Yunong Tian, Wenyuan Li, Lu Li, Dandan Li, Yulin Liu, Xiaoyue Tang, Yong Ye

**Affiliations:** ^1^ Department of Pharmaceutical Engineering, School of Chemistry and Chemical Engineering, South China University of Technology, Guangzhou, China, scut.edu.cn; ^2^ Department of Forestry, Jiangxi Environmental Engineering Vocational College, Ganzhou, China

**Keywords:** *Camellia oleifera* fruit shell, cellulose nanoparticles, histidine-rich protein, selective separation

## Abstract

Histidine deficiency has been associated with oxidative stress, inflammation, and metabolic diseases, compared with insecure use of a single amino acid supplement; histidine‐rich proteins are secure and can make up for the deficiency. Due to the low abundance of these proteins in food sources, there is a need for novel methodologies to effectively isolate histidine‐rich proteins. *Camellia oleifera* fruit shell is discarded as the waste, here used as raw material to make the oxidized cellulose (COC) with modification by aminothioureas (ATU), which further prepare a COC nanoparticle material with polyvinyl alcohol (PVA) combined with NiCoMnO_4_ nanoparticles by a directional freeze‐casting technique for the selective separation and purification of histidine‐rich proteins. The crystal structure, surface morphology, mechanical properties, and wettability were characterized and analyzed by XRD, SEM, compressive performance test, and contact angle test. The COC nanoparticles were used as a novel affinity material to investigate its adsorption properties for hemoglobin (BHb) and bovine serum albumin (BSA); the maximum adsorption capacity for BHb was determined to be 1970 mg/g, whereas the adsorption capacity for BSA was significantly lower, indicating that the material has selective adsorption for BHb. Moreover, the material demonstrated the ability to efficiently adsorb histidine‐rich proteins from different foods, and remain 90.2% of their initial adsorption capacity after 5 cycles, which is expected to be used in the enrichment of histidine‐rich proteins. This provides a new way for utilization of *C*. *oleifera* fruit shell and enrichment of histidine‐rich proteins as food nutrient fortifiers.

## 1. Introduction

Histidine (HIS) is an essential amino acid that must be obtained through dietary intake. It serves as a critical component of proteins and plays several vital roles in the body, including the promotion of metabolic processes, the production of white and red blood cells, ferritin, and hemoglobin, as well as the regulation of feeding behavior and energy metabolism. Additionally, HIS is involved in the synthesis of histamine and carnosine and contributes to the repair of cells and tissues within the body [1, 2]. HIS supplements can reduce oxidative stress and inflammation, improve metabolic health, support cognitive/mental health, resist colds and infections, diminish allergies, and protect the skin from UV radiation [3, 4]. Nevertheless, the use of a single amino acid supplement may lead to negative nitrogen balance, induce hypercholesterolemia and liver enlargement, and affect growth and even lead to mood disorders [5]. HIS deficiency can complement with HIS‐rich proteins. HIS accounts for about 2%–3% of most proteins (calculated by amino acid residues) and may reach 5% in HIS‐rich proteins, which include hemoglobin, myoglobin, HIS‐rich glycoproteins, histones, HIS‐rich calcium‐binding proteins, and polyfilament proteins [6]. However, the concentration of HIS‐rich proteins in food sources is relatively low. For instance, myoglobin is present at levels of 0.2–1.8 mg/g in beef and 0.6–4 mg/g in pork. Consequently, it is essential to enhance these proteins to satisfy specific dietary supplementation requirements [7, 8].

HIS‐rich proteins can be isolated and purified by immobilized metal affinity chromatography (IMAC), which is commonly used to purify recombinant proteins containing short affinity tags composed of multiple HIS residues [9, 10]. IMAC is mainly composed of solid phase matrix, chelating complexes, and metal ions. An ideal IMAC stationary phase has little interaction with the separated material and has good permeability and porous properties to allow macromolecules free access to and from it, besides being stable and reused [11]. The choice of stationary phase material largely depends on the purity of protein and efficiency of separation and purification. Currently, the fixed phase material used for protein separation is mainly particles and membrane, and particles are the most widely used. The particle has high adsorption capacity and separation accuracy, but because of the pore structure inside the particle medium, biological macromolecules usually need longer contact time to be fully adsorbed on the particle medium filled column, leading to poor accessibility and low processing efficiency [12]. In addition, when a large flux of liquid flows through, the particles will gradually accumulate dense, leading to problems such as large pressure increase and pore blocking, which greatly limits its application in the field of biological separation and purification [13]. The membrane separation matrix also has some inherent limitations, such as fiber relatively dense packing and relatively small pore size, which can lead to relatively large biomolecular diffusion barriers and high liquid flow resistance, resulting in low separation efficiency [14]. Consequently, there is a need for novel affinity materials to enhance the efficiency of protein separation and purification. As the most abundant natural macromolecular compound in nature, cellulose has good stability, biocompatibility, and mechanical strength, making it a suitable candidate for separation material [15]. *Camellia oleifera* fruit shell is a by‐product of *Camellia* oil seed processing with China′s annual output of 1 million tons, but it is discarded, leading to a great waste of resources [16]. Its cellulose content is about 28%, which has great utilization value [17]. However, cellulose usually gathers together in the state of the fiber bundle, and the active site and specific surface are greatly reduced, making it difficult to serve as affinity material directly.

Metal oxides such as CuO, NiO, and so on, have a specific affinity to HIS, thus, they can be used for its separation [18–20]. The metal oxide nanoparticles (NPs) can be used for rapid purification of HIS‐labeled proteins [21–23]. However, the NPs with small particle size are easy to agglomerate in solution, leading to the decrease in specific surface area and difficult reuse, which limits their application in protein industrial separation [24]. Therefore, in view of the difficulties of current HIS‐rich protein separation—such as mass resistance, low efficiency, low mechanical strength, poor stability, and nanometal oxide to aggregate [25]—a novel cellulose NP is designed as an affinity material to realize the specific and efficient separation and purification of HIS‐rich proteins.

*C*. *oleifera* fruit shell cellulose (COC) was prepared by combining citrate pretreatment, TEMPO (2,2,6,6‐tetramethylpiperidine‐1‐oxyl)‐NaBr‐NaClO catalytic oxidation and ultrasound treatment, to increase its active site and specific surface area, after activating –COOH by EDC (1‐ethyl‐3‐(3‐dimethylaminopropyl)) and NHS (N‐hydroxy succinimide); an amidation coupling reaction was performed to graft aminothiourea onto the surface of COC to obtain a modified nanocellulose (COC/ATU) to improve the ability for chelation of the metal ions. With physical crosslinking by adding polyvinyl alcohol (PVA) to increase its strength, the COC/ATU/PVA composite was formed and further combined with NiCoMnO_4_ NPs by coprecipitation to obtain the COC NPs. The material was characterized by x‐ray diffraction (XRD), SEM, compression performance test, contact angle test, TGA, surface morphology, mechanical properties, wettability, and thermal stability, and its separation and purification effects of HIS‐rich proteins from foods and reuse performance were investigated. In conclusion, the innovation and benefits of this study are encapsulated in the following elements: (i) The employment of *C*. *oleifera* fruit shell waste as a raw material offers a sustainable and cost‐effective source of cellulose. (ii) The synthesized COC NP material incorporates a biocompatible and stable cellulose framework, a chelating ligand, and the specific affinity of NiCoMnO_4_ NPs, with the objective of achieving efficient and selective capture of HIS‐rich proteins. (iii) This composite material demonstrates a high adsorption capacity (1970 mg/g for bovine hemoglobin [BHb]) and notable selectivity over nontarget proteins (bovine serum albumin [BSA]), as evidenced in the manuscript. (iv) The material exhibits excellent reusability, retaining 90.2% of its capacity after five cycles, which is essential for practical applications in the enrichment of HIS‐rich proteins from foods as nutrient fortifiers.

## 2. Materials and Methods

### 2.1. Materials

Ni(CH_3_CO_2_)_2_·4H_2_O and Mn(CH_3_CO_2_)_2_·4H_2_O were bought from Qianhui Chemical Reagents Company (Guangzhou, China); Co(CH_3_CO_2_)_2_·4H_2_O, imidazole, BSA, and BHb were purchased from Shanghai Macklin Biochemical Co. Ltd (Shanghai, China). Aqueous ammonia and other reagents were provided by Guangzhou Chemical Reagent Factory (Guangzhou, China). Meat and milk are bought from the supermarket in Guangzhou, China.

### 2.2. Preparation of NiCoMnO_4_ NPs

The NiCoMnO_4_ NPs were prepared by coprecipitation method [24]. In brief, 1 mL of 0.2‐M Ni(CH_3_CO_2_)_2_·4H_2_O, Mn(CH_3_CO_2_)_2_·4H_2_O, and Co(CH_3_CO_2_)_2_·4H_2_O were mixed into 30 mL of deionized water, respectively. Then, the mixture was sonicated under an ice bath in a power of 300 W for 20 min; meanwhile, 2 mL of aqueous ammonia were added as a coprecipitation agent, resulting in a distinct color change from deep green to gray. Subsequently, the mixture was placed into a reactor at 160°C for 3 h after being stirred (500 rpm) at 80°C for 3 h. The yielded precipitate was collected after centrifugation at 6000 rpm for 10 min, then dried overnight at 70°C. Finally, the NPs were obtained after calcination in a muffle furnace (Wuhan Jisi Apparatus Company, Wuhan, China) at 500°C with a heating rate of 2°C/min for 2 h.

### 2.3. Preparation of *C*. *oleifera* Cellulose

The cellulose nanofibers were produced from the fruit shell of *C*. *oleifera* and prepared by an oxidation method according to the reference after several modifications [26]. The fruit shell powder of *C*. *oleifera* was added in 1.5% (*w*/*v*) sodium chlorate solution at a mass/volume ratio of 1:10 and constant stirring in 70°C for 2 h. The white residue was collected and washed to neutral, and then added in 1‐mol/L NaOH solution and stirred for 2 h (6°C). Following washing and filtration, the product was submerged in distilled water overnight, and dried in vacuum condition. Subsequently, this product was suspended in a 8% (*v*/*v*) HCl aqueous solution with stirring for 24 h. The cellulose was obtained after filtration and washing with distilled water.

A total of 10 g of the cellulose was dispersed in 1 L of deionized water at room temperature, and then, 0.16 g of TEMPO and 1 g of NaBr were slowly added and dissolved. With dropwise adding 100 mL of a 9% NaClO solution, the pH was monitored and adjusted to 10 by 0.5‐mol/L NaOH to maintain the reaction for 12 h. Finally, 10 mL of ethanol was added to terminate the reaction; the mixture (COC) was stored at 4°C for future use.

### 2.4. Preparation of Aminothiourea Modified COC

Aminothiourea modification can not only improve activity of some compound [27], but also enhance the binding affinity of cellulose to metal oxides, the COC were modified with aminothiourea via carbodiimide reaction. Briefly, 200 g of the COC was mixed with 1.5 mmol of EDC and 1.5 mmol of NHS to activate the carboxyl groups. The binding reaction took place and was maintained for 24 h after 10 mL of a 0.4‐mol/L aminothiourea solution was added to the mixture. The reactant was centrifuged at 3500 rpm for 10 min, and the supernatant was dialyzed in distilled water with a membrane of molecular weight 10,000‐Da cutoff for 24 h, followed by freeze‐drying to obtain the COC derivative (COC/ATU).

### 2.5. Preparation of COC NPs

**Figure 1:**
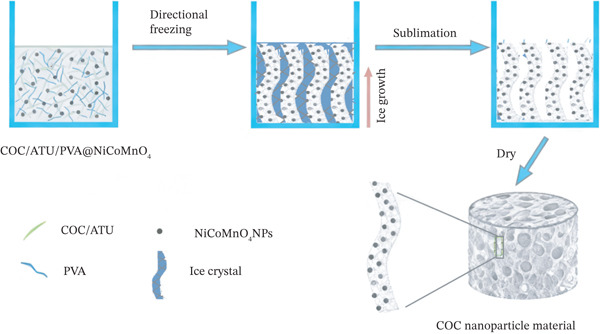
Preparation process of COC nanoparticle material.

The preparation process of COC NPs is illustrated in Figure 1. Firstly, 0.5‐wt% COC/ATU suspension in distilled water and NiCoMnO_4_ NPs were mixed well and ultrasonicated on ice bath at 400 W for 10 min. Afterwards, the mixture was coupled with 2‐wt% PVA for 1 h at room temperature. During a liquid nitrogen directional freeze‐casting drying process, COC NPs were kept in regular porous shape. Different mass ratios (9:1, 8:2, and 7:3) of COC/ATU and NiCoMnO_4_ NPs were investigated in the preparation for the optimization.

### 2.6. Structural Analysis

The chemical structure, crystallinity, morphology, and surface properties of the samples were characterized using a suite of techniques. Fourier transform infrared (FTIR) spectra were acquired on a Bruker VERTEX 70 spectrophotometer within the range of 4000–400 cm^−1^ using KBr pellets. XRD patterns were recorded on a PD 3500 diffractometer (Dandong Tongda) with Cu K*α* radiation. The surface morphology and elemental composition were examined using an SU8820 ultrahigh‐resolution scanning electron microscope (Hitachi) equipped with an EDS detector. Surface elemental speciation was quantified by x‐ray photoelectron spectroscopy (XPS) on a Kratos AXIS NOVA spectrometer. The hydrophobicity was evaluated by measuring water contact angles with an SDC350 tester (Sindi); the reported values are the average of measurements taken at three distinct locations on the material surfaces.

### 2.7. Compressive Performance Experiments

The compression performance of materials was measured by a universal compressor (Metes Industrial Systems [China] Co. Ltd, Shanghai, China). Samples (20 mm in diameter and 10 mm in height) were compressed into 20% of the initial size with a speed of 10 mm/min. The stress–strain curve during loading and unloading cycles was recorded.

### 2.8. HIS‐Rich Proteins Adsorption Experiments

The COC NP materials were used as the affinity separation materials. In order to investigate their adsorption properties for HIS‐rich proteins, BHb, and BSA, the absorption experiments were carried out in a typical process. The materials (0.1 g) were immersed in 20 mL of PBS (20 mmol/L, pH 7.2) for 30 min, and then, 100 mL of BHb (1 mg/mL) and BSA (1 mg/mL) were added together in the system for absorbing 30 min. BSA proteins were eluted from the materials by PBS solution (20 mmol/L, pH 7.2), whereas the specific BHb proteins were eluted by imidazole solution dissolved in PBS (20 mmol/L, pH 7.2) subsequently. The collected eluents were used to evaluate the absorption capacity of BSA and BHb proteins. In order to find out the optimal experimental conditions, pH (5.0–10.0), absorption time (1–60 min), and original concentrations of BSA and BHb (0.1, 0.2 mg/mL, 0.4 mg/mL, 0.6 mg/mL, 0.8 mg/mL, and 1.0 mg/mL), were also performed.

The BSA and BHb concentration in solutions was determined by HPLC (Agilent 1026, Agilent Technologies, Santa Clara, California, United States). Analysis conditions were as follows: Mobile phase A was 0.1% TFA aqueous solution; mobile phase B contained 0.01% TFA acetonitrile solution; flow rate was 1.5 mL/min; column temperature was 40°C; the column used was Hypersil BDS C8, the sample volume is 20 *μ*L; the UV detector wavelength is 280 nm. The protein concentration of the samples was calculated based on the protein concentration–peak area standard curve.

The BSA and BHb concentration differences before and after absorption were used to evaluate the absorption capacity of the materials; the capacity is calculated in Equation (1)

(1) Q=c0−ct×V0×1000m

where *Q*, *V*_0_, *C*_0_, *C*_*t*_, and *m* represent absorption capacity (mg/g), initial volume (mL), proteins concentration before absorption (mg/mL), proteins concentration after absorption (mg/mL), and weight of materials samples (g).

### 2.9. Reusable Performance Experiments

The BHb proteins were absorbed and eluted for five times repetition, as the same as the former methods to evaluate the reusable performance of the material.

### 2.10. HIS Proteins Enrichment From Foods by the Material

A total of 10 g of fresh pork, beef, pig blood, ox blood, and milk were collected and homogenated with 200‐mL PBS (20 mmol/L, pH 7.2), respectively, then centrifugated at 5000 rpm for 20 min. The supernatant was precipitated by saturated ammonium acetate, then redissolved in PBS (20 mmol/L, pH 7.2) solution and adsorbed by the COC NP materials with above method. Total protein content was determined by an UV‐Vis spectrophotometer (SHIMADZU UV‐2550, Japan) at 554 nm [28]. HIS content before and after separation was determined as follows: 5 mL of the sample solution was added to 1‐mL NH_3_ H_2_O‐NH_4_Cl buffer solution, 1‐mL 0.045 mg/mL p‐aminobenzene sulfonic acid, and 1 mL 10% sodium nitrate solution, mixed for 20 min, then, we determined the absorbance at 530 nm [29]. HIS content (*y*) was calculated based on the standard curve *y* = 0.9.9302*x* + 0.0351 (*r* = 0.9839), which showed a good linear relationship in the concentration range of 0.01 to 0.1 mg/mL.

### 2.11. Statistical Analysis

Data from all experiments were collected in triplicate and then presented in the form of means ± standard deviation. One‐way analysis of variance (ANOVA) was used to analyze the data from all experiments at the significance level of *p* < 0.05.

## 3. Results and Discussion

### 3.1. FTIR Spectra of COC, Oxidized, and Modified COC

**Figure 2:**
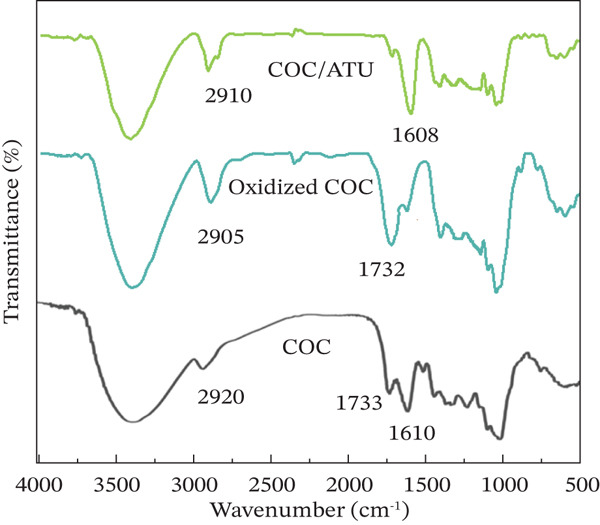
FTIR spectra of COC, oxidized COC, and COC/ATU.

To investigate the group differences in *C*. *oleifera* fruit shell cellulose before and after TEMPO oxidation and aminosourea modification, FTIR analysis of COC, oxidized COC, and modified COC (COC/ATU) were performed in the range of 500–4000 cm^−1^, as shown in Figure 2. For COC, the band at 3600–3100 cm^−1^ indicated the strong and relatively broad –OH vibration absorption, due to the intramolecular and intermolecular bonds in the cellulose structure and the free‐OH groups. The absorption band at 1200–1000 cm^−1^ corresponded to the expansion vibration of C‐O; 2920 cm^−1^ was the expansion vibration absorption of C‐H. Compared with COC, the main change in oxidized COC is the characteristic C = O absorption band at 1732 cm^−1^, indicating that TEMPO oxidation successfully introduced –COOH in cellulose. It existed a strong –OH stretching vibration absorption band at 3600–3100 cm^−1^, indicating that a large amount of –OH remains in oxidized cellulose, further indicating the selective –OH oxidation of the TEMPO‐NaBr‐NaClO system. For COC/ATU, the characteristic carboxyl peak at 1732 cm^−1^ disappeared, confirming the consumption of –COOH groups. Meanwhile, a significant new absorption band emerged at 1608 cm^−1^. Rather than a C = N band, this peak was assigned to the Amide II band (primarily N‐H bending and C‐N stretching vibrations) associated with the newly formed amide linkage, combined with the N‐H scissoring vibrations from the aminothiourea moiety. The typical Amide I band was considered to be merged within the broad hydroxyl/adsorbed water bending vibration. These spectral changes confirmed that aminothiourea has been successfully grafted onto the oxidized COC via amide bonds.

### 3.2. XRD Character of COC NPs

**Figure 3:**
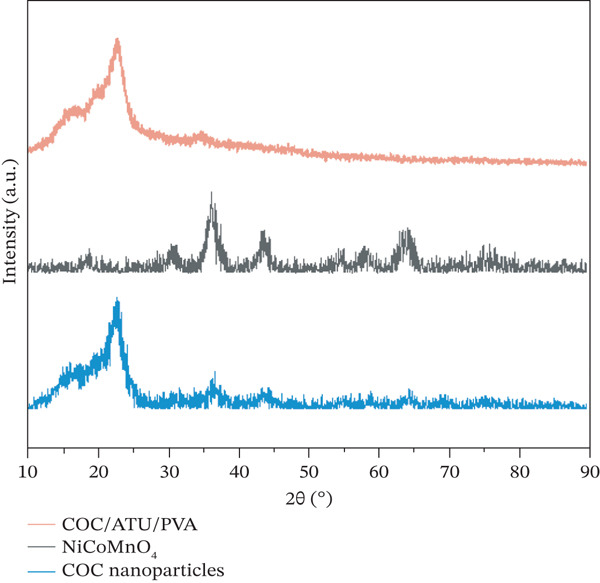
XRD patterns of COC/ATU/PVA, NiCoMnO_4_, and COC nanoparticles.

XRD patterns of three samples (COC/ATU/PVA, NiCoMnO_4_, and COC NPs) were illustrated in Figure 3. Three typical diffraction peaks at 2*θ* = 16.6^°^, 22.6^°^, and 34.7^°^ in the COC/ATU/PVA were corresponded to the (10$\overset{\wedge }{1}$), (002), and (004) reflections of cellulose Type I, indicating an integral cellulose crystal structure of the composite materials, whereas diffraction peak at 2*θ* = 19.9^°^ was corresponded to the reflections of PVA. In addition, characteristic peaks of NiCoMnO_4_ were founded in the COC NPs, peaks at 2*θ* = 18.7^°^, 30.5^°^, 36.3^°^, 37.5^°^, 43.6^°^, 54.7^°^, 58.2^°^, and 63.9^°^, corresponding to (111), (220), (311), (222), (400), (422), (511), and (440) crystal faces of NiCoMnO_4_ (JCPDS No. 20‐0781). Characteristic peaks of COC/ATU, PVA, and NiCoMnO_4_ founded in the COC NPs suggest that the NiCoMnO_4_ NPs were loaded into the COC/ATU/PVA material.

### 3.3. Surface Morphology of COC NPs

**Figure 4:**
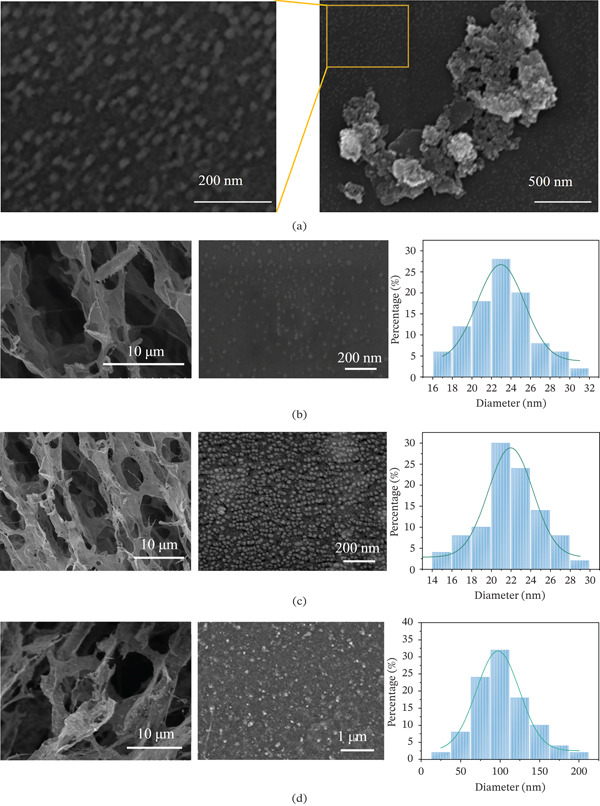
(a) Amplified SEM images of NiCoMnO_4_ NPs and (b–d) SEM and particle size distribution of COC nanoparticles in different mass ratios of COC/ATU and NiCoMnO_4_: (b) 9:1, (c) 8:2, and (d) 7:3.

Surface morphology was observed by SEM. As illustrated in Figure 4a, the NiCoMnO_4_ NPs appeared spherical in shape with an average particle size less than 30 nm, whereas some of the NPs agglomerated together forming irregular masses. The internal structure and surface morphology of the COC NPs with different mass ratios (9:1, 8:2, and 7:3) of COC/ATU and NiCoMnO_4_ were detected by SEM; the average particle sizes of NiCoMnO4 NPs on the surfaces were analyzed by Image‐Pro Plus software. As illustrated in Figure 4b–d, the material presented a honeycombed structure with numerous pores. When the mass ratio was 9:1, the particle size predominantly fell within the range of 21–25 nm. And at a mass ratio of 8:2, the particle size was primarily distributed between 20 and 24 nm. Notably, when the mass ratio was further increased to 7:3, particle aggregation was observed, resulting in an increase in particle size to approximately 100 nm.

### 3.4. EDS Elemental Mapping Analysis of COC NPs

**Figure 5:**
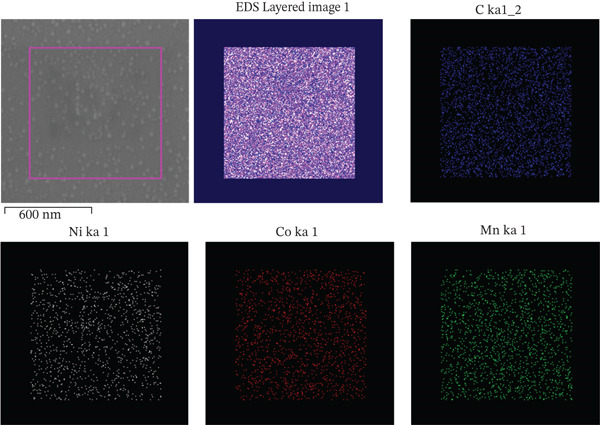
Mapping of COC nanoparticle material.

Figure 5 is an EDS elemental mapping of COC NPs. It confirms the uniform distribution of the three elements Mn, Co, and Ni on the carbon skeleton.

**Figure 6:**
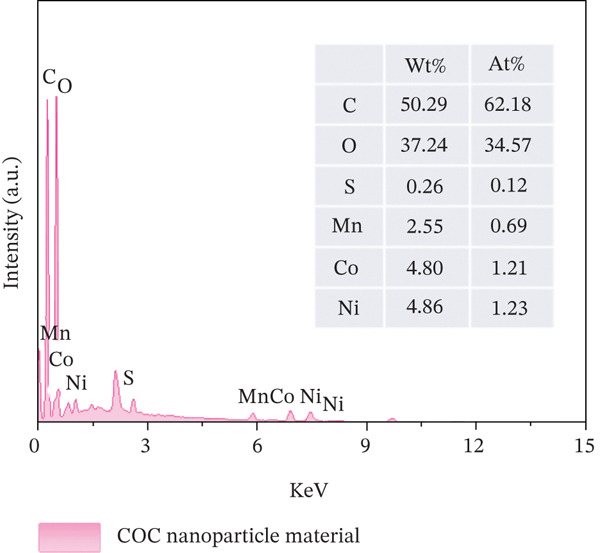
EDS spectra of COC nanoparticle material.

As shown in Figure 6, the energy dispersive spectra revealed that COC NPs contained at least the C, O, S, Mn, Co, and Ni elements. The element content of Mn, Co, and Ni was 2.55%, 4.80%, and 4.86%, respectively, and the relative content ratio was about 1:2:2. It can be concluded that the COC NPs prepared by this method have a high loading rate of NiCoMnO_4_ NPs.

### 3.5. XPS Analysis of COC NPs

**Figure 7:**
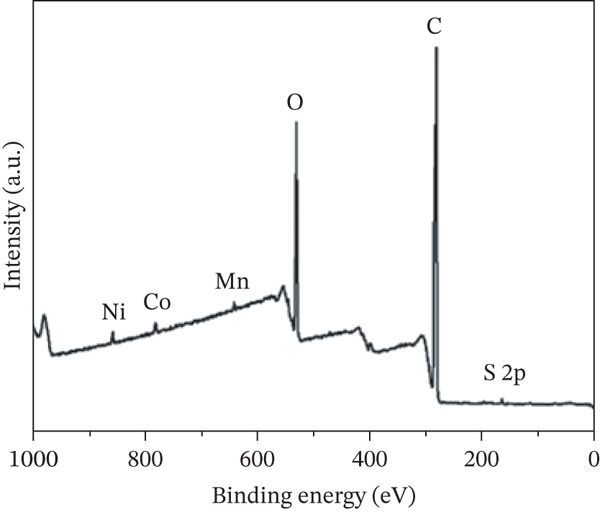
XPS spectra of COC nanoparticle material.

To further examine the surface chemical composition and verify the successful incorporation of the functional components, XPS was conducted. As demonstrated in Figure 7, the composite material predominantly consists of the elements C, O, S, Mn, Co, and Ni. The prominent peaks associated with C and O are attributed to the structural framework of cellulose and PVA. Notably, the distinct S 2p peak was observed, which confirmed the successful grafting of the aminothiourea functional groups onto the oxidized cellulose surface. Moreover, the characteristic peaks of Ni, Co, and Mn were clearly detected in the spectrum. The result forcefully proved that the NiCoMnO_4_ NPs were effectively loaded and well‐exposed on the outermost surface of the COC/ATU/PVA matrix, which was in excellent agreement with the EDS mapping results, collectively verifying the successful fabrication of the target COC NP affinity material.

### 3.6. BET and Pore Size Distribution of COC NPs

**Figure 8:**
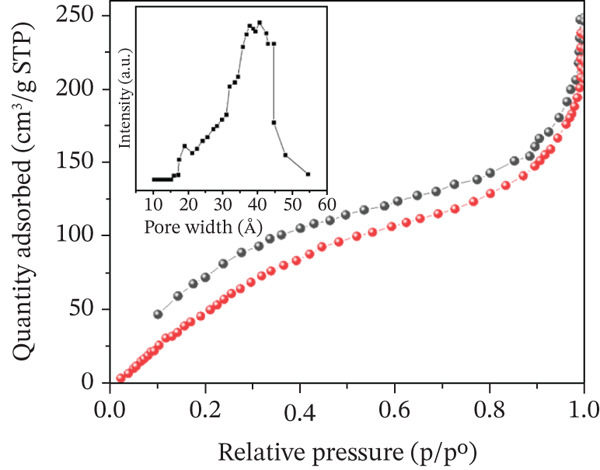
N_2_ adsorption–desorption isotherms and pore size distribution of COC nanoparticle materials; red dots represent adsorption and black dots represent desorption.

As shown in Figure 8, COC NPs exhibited a typical Type IV isotherm with a distinct hysteresis loop, which perfectly confirmed the existence of an abundant mesoporous structure within the material skeleton. Furthermore, the pore size distribution revealed a prominent mesopore peak centered at approximately 40–42 Å and the BET specific surface area of the material was 65.2 m^2^/g. The mesoporous structure in the material provided an excellent platform for the uniform dispersion of NPs, preventing their severe agglomeration and maximally exposing the Ni, Co, and Mn affinity sites to the surrounding fluid. Combined with SEM, this synergistic effect of “macropores for diffusion + mesopores for loading and binding” essentially endowed the material with highly accessible and abundant specific binding sites for capturing HIS‐rich proteins.

### 3.7. Compressive Performance of COC NP Material

**Figure 9:**
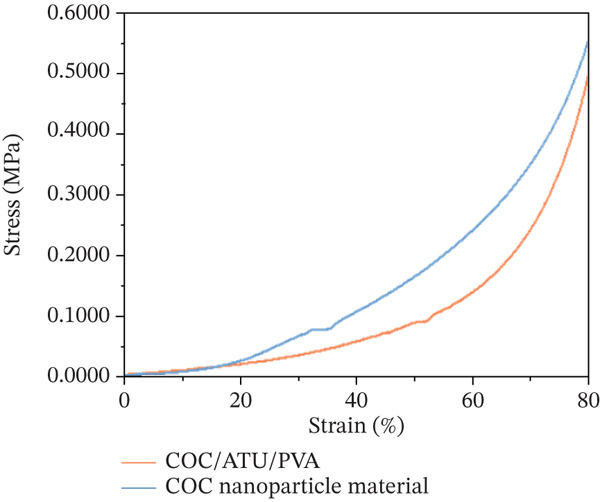
Stress–strain curves of COC nanoparticle materials.

The stress–strain curves of COC/ATU/PVA and COC NP material are compared in Figure 9. It can be seen that the composite material loaded with NiCoMnO_4_ NPs exhibited a higher compressive stress than the COC/ATU/PVA (0.5508 MPa vs. 0.4943 MPa) at 80% strain, and the compressive modulus of the materials increased from 0.2738 to 0.6221 MPa after loading with NiCoMnO_4_ NPs, indicating a better compressive properties than the COC/ATU/PVA. The explanation can be linked to the formation of strong hydrogen bonds between COC/ATU, PVA, and NiCoMnO_4_, which enhances the stability of the three‐dimensional network.

### 3.8. Wettability of the COC NP Material

**Figure 10:**
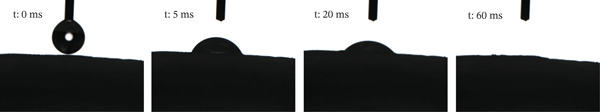
Water contact angle of COC nanoparticle material.

A promising absorbing material for proteins requires a good wettability which can efficiently absorb and transport proteins in buffered solution efficiently. The water contact angles on the internal and external surface of COC NP material were measured to evaluate the water transportation ability. As shown in Figure 10, a drop of water (2 *μ*L) can be completely absorbed within 60 ms, indicating a better hydrophilicity, which is conducive to water transport during the protein absorption process.

### 3.9. HIS‐Rich Protein Absorption Ability

The COC NP material was used in HIS‐rich protein affinity absorption as an affinity separation material. BHb, as a HIS‐rich protein, and BSA as a common protein, were used to evaluate the separation effectiveness of COC NP material. Separation conditions such as pH value, absorbing time, and original concentrations of BSA and BHb were compared between the two proteins.

### 3.10. pH Value

**Figure 11:**
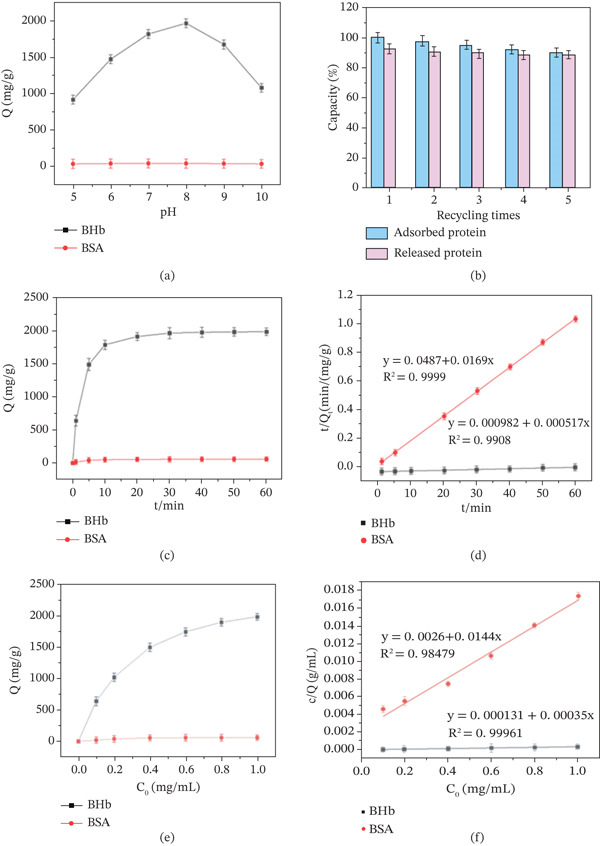
(a) Effect of pH on adsorption of BHb and BSA, (b) adsorption capacity and recovery of COC nanoparticle material for BHb under different recycling times, (c) BHb and BSA adsorption kinetic curve, (d) kinetic fitting of the COC nanoparticle material adsorbing BHb and BSA, (e) BHb and BSA adsorption isotherms, and (f) linear fitting equation of Langmuir.

As shown in Figure 11a, pH value had no significant influence on the absorption capacity of BSA, whereas the absorption of BHb first increased as the pH value raised, reaching a maximum at pH 8, and then decreased. The explanation can be linked to the selective absorption: HIS‐rich hemoglobin can specifically interact with NiCoMnO_4_ NPs loaded on the surface of the composite material to achieve selective adsorption, whereas BSA with low contents of HIS has poor interaction resulting in a low absorption capacity.

### 3.11. Absorbing Time

The adsorption kinetic curves of COC NP material for BHb and BSA are shown in Figure 11c. The adsorption quantity for BSA was not significantly affected by time, whereas the BHb adsorbed amount was significantly influenced by time. The adsorption amount for BHb increased rapidly in the first 10 min and then reached the adsorption equilibrium within 30 min with the adsorption amount of 1970 mg/g. Table 1 summarizes the adsorption capacity of different adsorbents for hemoglobin. It can be seen that the adsorption capacity of COC NP for hemoglobin is significantly higher than that of other adsorbents. This is because more binding sites on the surface of the material and a higher protein concentration lead to the rapid increase of adsorption amount in the early stage. This result indicates that the COC NP material can achieve rapid and efficient adsorption for BHb. Figure 11d shows the nonlinear fitting kinetic models of the COC NP material adsorbing BHb and BSA. Results showed that the correlation coefficients (*R* ^2^) of pseudosecond order kinetic model of the BHb and BSA adsorbed onto the COC NP material were 0.9999 and 0.9908, which can be well described by the pseudosecond order model. The results suggest that the adsorption process of BHb and BSA on the COC NP material kinetics data may be influenced by coordination interactions.

**Table 1:** Adsorption capacities for hemoglobin of various adsorbents.

Adsorbent	Adsorption capacity (mg g^−1^)	Ref.
NiFe_2_O_4_‐PAA‐Cu^2+^	783.5	[30]
Fe_3_O_4_@MoO2∩C‐Ni	675.7	[31]
MnFe_2_O_4_@SiO_2_@Er_2_O_3_	238.2	[32]
MagGO@BR‐IMA‐Cu^2+^	378.6	[33]
ZIF‐GO_4_ composite	433	[34]
GO@SiO_2_@C‐Ni	783	[35]

### 3.12. Initial Concentrations

According to the results of adsorption kinetics experiments, the adsorption time was set at 30 min. By changing the initial protein concentration (0.1 ~ 1.0 mg/mL) of the solution, adsorption experiments in various initial concentrations were conducted on COC NP material in PBS buffer solution at pH 8. Figure 11e shows the isotherm adsorption curves of COC NP material for BHb and BSA. The adsorption for BSA increased slightly by the increase of initial concentration, reaching saturation with the initial concentration at approximately 0.4 mg/mL, and the adsorption amount was very low. In contrast, the adsorption amount of BHb increased rapidly with increasing initial concentration, reaching the maximum with the initial concentration of 1 mg/mL. Figure 11f shows the isotherm adsorption curves of COC NP material for BHb and BSA. Based on the experimental results, a composite Langmuir adsorption model for the adsorption of BHb by COC NP material was inferred, with an *R* ^2^ value of 0.9996.

### 3.13. Reuse Performance of COC NP Materials

To investigate the reuse performance of COC NP material, the material after adsorbing BHb was washed with eluent and then reused for the adsorption of BHb. As shown in Figure 11b, after five cycles of adsorption–elution, the adsorption capacity of COC NP material for BHb remained 90.2% and decreased by about 9.8%. Meanwhile, the recovery rate was 92.7% during the first use, and only dropped by 3.9% after five cycles of reuse, showing a less decline. Reuse experiments indicate that COC NP material has good recyclable performance.

### 3.14. Effect of HIS‐Rich Proteins Enrichment by COC NP Materials

The specific enrichment of HIS‐rich proteins by the COC NP material was fundamentally governed by coordination chemistry based on the Lewis acid–base theory. The transition metal ions (Ni, Co, and Mn) presented on the surface of the composite material exhibited vacant d‐orbitals, thereby allowing them to function as potent Lewis acids (electron acceptors). Conversely, the HIS residues abundantly exposed on target proteins contain imidazole rings. The unprotonated imine nitrogen atom within this imidazole ring possessed a lone pair of electrons, acting as a potent Lewis base (electron donor). Upon contact, these imine nitrogen atoms specifically donated their electron pairs to the empty d‐orbitals of the metal ions, forming highly stable coordination bonds. Nontarget proteins lacking sufficient accessible HIS residues experience only weak physical interactions and were subsequently washed away. During the elution process, the addition of a high concentration of free imidazole introduced a competitive binding mechanism. Because free imidazole shared the exact same functional group as the HIS side chain, it violently competed for the transition metal sites, effectively displacing the coordinated target proteins and allowing for their recovery and the regeneration of the affinity material. The COC NP material served as the separation medium to purify the HIS‐rich proteins from meat, blood, and milk. The results were shown in Table 2. HIS content significantly increased after adsorption separation by the COC NP material treating different sources of proteins. The lower the HIS content in raw materials, the more the adsorbed HIS increased; the increase ranged from 1.57 fold to 18.53 fold with different sources of proteins.

**Table 2:** Enrichment results of histidine‐rich proteins in different sources by the COC nanoparticle material.

Source	His content before enrichment (mg/g protein)	His content after enrichment (mg/g protein)	Increase (fold)
Beef	0.58 ± 0.12	11.33 ± 1.74	18.53
Pork	1.13 ± 0.26	14.28 ± 2.53	11.64
Pig blood	86.36 ± 7.62	352.57 ± 21.28	3.08
Ox blood	127.38 ± 19.32	464.83 ± 24.62	2.65
Milk	224.63 ± 36.27	576.63 ± 45.42	1.57

## 4. Conclusion

*C*. *oleifera* fruit shell can be an ideal raw material to make cellulose, with oxidation by TEMPO‐NaBr‐NaClO and modification by aminothiourea; the modified COC is obtained. Following the loading of NiCoMnO_4_ NPs onto COC/ATU/PVA and subsequent directional freeze‐casting drying, the COC NP material is formed, and its microstructure, compressive properties, thermal stability, and wetting and adsorption properties of HIS‐rich proteins have been investigated.

1. Based on the results obtained from XRD, SEM, and EDS, COC/ATU and NiCoMnO_4_ NPs have been successfully prepared. The NiCoMnO_4_ NPs exhibit an average particle size of approximately 22 nm and demonstrate effective loading and uniform dispersion on the surface of COC/ATU/PVA. This dispersion effectively addresses the issue of NiCoMnO_4_ NP agglomeration.
2. The compression performance assessment indicates that COC NPs exhibit a compressive stress of 0.5508 MPa and a compression modulus of 0.6221 MPa, representing an improvement over the COC/ATU/PVA composite and demonstrating favorable compression characteristics. Additionally, the contact angle analysis reveals that the COC NP material possesses excellent wettability. These properties establish a robust foundation for the application of COC NPs as an affinity medium for the efficient isolation and purification of target proteins.
3. The optimal adsorption conditions of BHb by COC NP material were investigated. When the adsorption time was set to 30 min and the initial concentration of BHb was 1 mg/mL, the maximum adsorption capacity of the materials reached 1970 mg/g. Furthermore, the COC NP material demonstrated lower adsorption affinity for BSA while exhibiting a significantly higher adsorption capacity for BHb. These findings suggest that COC NP material is an excellent medium for the separation of hemoglobin.
4. The material relies on the complex interaction of Ni, Co, Mn, and HIS residues on the surface of the proteins to realize the adsorption of HIS‐rich proteins from different foods, and can be applied to the effective enrichment of the HIS‐rich proteins. This provides a new way for utilization of *C*. *oleifera* fruit shell and enrichment of HIS‐rich proteins as food nutrient fortifiers.

## Author Contributions

**Yunong Tian:** conceptualization, writing – original draft preparation. **Wenyuan Li:** methodology, software, writing – original draft preparation. **Lu Li:** methodology, software. **Dandan Li:** investigation, resources. **Yulin Liu:** investigation, resources. **Xiaoyue Tang:** validation, visualization. **Yong Ye:** writing – review and editing, supervision, project administration, funding acquisition. **Yunong Tian** and **Wenyuan Li** contributed equally to the manuscript.

## Funding

This study was supported by the Jiangxi Provincial Natural Science Foundation (Grant Number 20244BAB28061), Qingyuan City Science and Technology Plan Projects (2023DZX007), and the Science and Technology Plan Project of Guangzhou City (2024B03J1269).

## Disclosure

All authors have read and agreed to the published version of the manuscript. A preprint has previously been published [36], and the discarded *Camellia oleifera* fruit shell has been found as a source of cellulose to synthesize the new complex COC nanoparticle material in this article, and expanded its use in the separation of histidine‐rich proteins from different foods.

## Conflicts of Interest

The authors declare no conflicts of interest.

## Data Availability

All data generated or analyzed during this study are included in this article.
